# The Effect of Alkali and Ce(III) Ions on the Response Properties of Benzoxazine Supramolecules Prepared via Molecular Assembly

**DOI:** 10.3390/molecules17010511

**Published:** 2012-01-05

**Authors:** Attaphon Kaewvilai, Sawittree Rujitanapanich, Worawat Wattanathana, Chatchai Veranitisagul, Songwut Suramitr, Nattamon Koonsaeng, Apirat Laobuthee

**Affiliations:** 1 Department of Materials Engineering, Faculty of Engineering, Kasetsart University, Bangkok 10900, Thailand; Email: kaewvilai@hotmail.com; 2 Chemistry Program, Faculty of Science and Technology, Phranakhon Rajabhat University, Bangkok 10220, Thailand; Email: sawittree_rj@yahoo.com; 3 Department of Chemistry, Faculty of Science, Kasetsart University, Bangkok 10900, Thailand; Email: w_worawat@hotmail.com (W.W.); fsciswsm@ku.ac.th (S.S.); fscinmk@ku.ac.th (N.K.); 4 Department of Materials and Metallurgical Engineering, Faculty of Engineering, Rajamangala University of Technology Thanyaburi, Pathumthani 12110, Thailand; Email: veranitisagul.c@gmail.com; 5 Center of Advanced Studies in Industrial Technology, Faculty of Engineering, Kasetsart University, Bangkok 10900, Thailand

**Keywords:** benzoxazine monomers, supramolecules, Pedersen’s technique, complexation, molecular assembly

## Abstract

A series of benzoxazine monomer supramolecules with different substituted groups on their benzene ring was prepared with a Mannich reaction and characterized by FTIR, ^1^H-NMR and MS. The obtained products were 3,4-dihydro-3-(2’-hydroxyethylene)-6-methyl-2H-benzoxazine (**BM1**), 3,4-dihydro-3-(2’-hydroxyethylene)-6-ethyl-2H-benz-oxazine (**BM2**), and 3,4-dihydro-3-(2’-hydroxyethylene)-6-methoxy-2H-benzoxazine (**BM3**). The efficiency of alkali metal ion extraction from the products was determined with Pedersen’s technique, while the complexation of the Ce(III) ion was confirmed by the Job’s and the mole ratio methods. The evidence of complex formation between benzoxazine monomers and Ce(III) ions was obtained with FTIR and a computational simulation. Single phase ceria (CeO_2_) as observed with XRD was successfully prepared by calcinating the Ce(III)-benzoxazine monomer complexes at 600 °C for 2 h. In addition, the geometry of the ceria nanoparticles confirmed by TEM is spherical, with an average diameter of 10‑20 nm.

## 1. Introduction

Supramolecules are known as specific structures, usually in two- or three-dimensions, obtained by linking a series of molecules. Their properties are completely different from the individual components [1,2,3,4,5]. The molecular interactions between supramolecules and guests to form host-guest compounds or inclusion compounds are induced by non-covalent interactions or secondary forces, such as van der Waals, dipole-dipole interaction, hydrogen bonding, *etc* [1,2,3,4,5]. Therefore, novel supramolecules with specific functional groups for the desired properties have been designed and proposed as both molecular assembly and cyclic structures [1,2,3,4,5].

Chirachanchai *et al.* originally proposed some novel supramolecules, benzoxazine oligomers, as ion extraction materials [6]. According to this idea, the guest-responsive ability of the benzoxazines resembled to repeating unit of calixarenes and acyclic *para*-substituted phenol-formaldehyde oligomers was investigated [6]. 

To understand the properties of benzoxazine supramolecules, systematical studies of the inclusion phenomena of a series of benzoxazine molecules have been performed [6,7,8,9,10,11,12,13]. As expected from the molecular designs, *p*-substituted benzoxazine monomers have been used as a starting materials in ring opening reactions to obtain novel benzoxazine molecules of either the cyclic or non-cyclic type [6,7,8,9,10,11,12,13]. Although since 1944 benzoxazine monomers have been simply prepared by a Mannich reaction from phenol, formaldehyde and primary amine sa originally proposed by Holly and Cope [14], there were few studies about benzoxazine monomers as supramolecules [15]. Based on the molecular structures of the benzoxazine monomers, it turned out to be an interesting objective to determine the metal ion responsive properties of the *p*-substituted benzoxazine monomers. The aim of this work was, therefore, to prepare benzoxazine monomers with a Mannich reaction and to investigate their ion extraction abilities with alkali metal ions with Pedersen’s technique [16,17]. 

In our related work, the complex method has been focused to prepare highly pure and homogeneous ceria for applications in the solid support catalyst for electrolytes for solid oxide fuel cells, methane steam reforming and hydrogenation [18,19,20]. In this work, the benzoxazine monomers used as novel ligands for rare earth ions such as Ce(III) were also qualitatively and quantitatively studied by the Job’s and molar ratio methods. The evidence of the complex formation between benzoxazine monomers and Ce(III) ions was clarified with FTIR and a computational simulation. In addition, the obtained complexes were further applied as precursors for the preparation of ceria powder. 

## 2. Results and Discussion

### 2.1. Preparation of Monomers by the Mannich Reaction

**Scheme 1:**
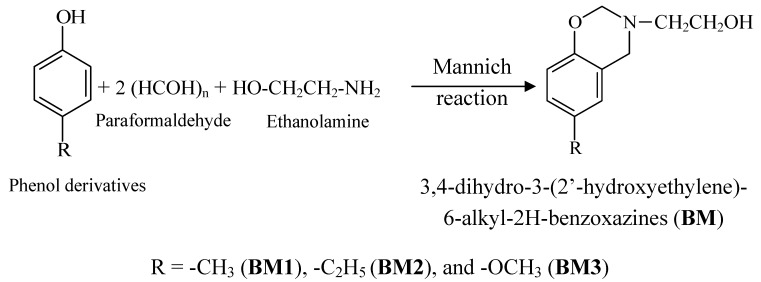
Preparation of benzoxazine monomers by Mannich Reaction.

Benzoxazine monomers (3,4-Dihydro-3-(2’-hydroxyethylene)-6-alkyl-2H-benzoxazines, **BM**) were prepared by a Mannich reaction as shown in Scheme 1.

**Figure 1:**
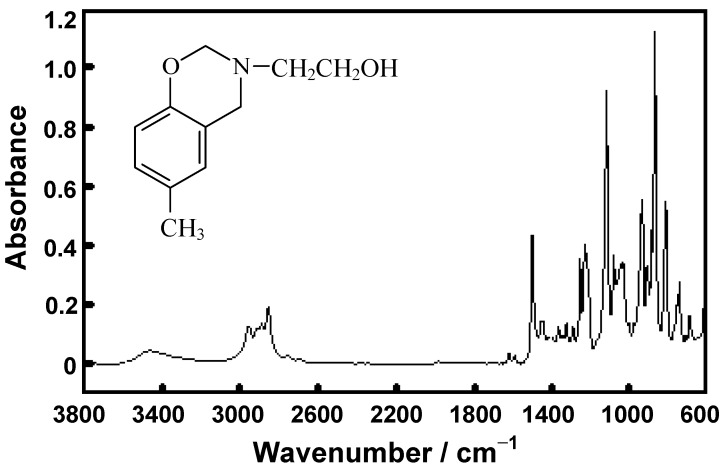
FTIR spectrum of **BM1**.

To confirm the successful preparation of benzoxazine monomers by a Mannich reaction, the reactions were examined by FTIR, ^1^H-NMR and MS. Typical results as exemplified by **BM1** were as follows: as shown in Figure 1, the FTIR spectrum of **BM1 **shows a broad peak around 3600–3200 cm^−1^ due to the alcohol hydroxyl group. There are also three characteristic peaks for **BM1 **found at 1500, 1226, and 1126 cm^−1^ belonging to trisubstituted benzene, C-N stretching, and C-O-C stretching, respectively.

**Figure 2:**
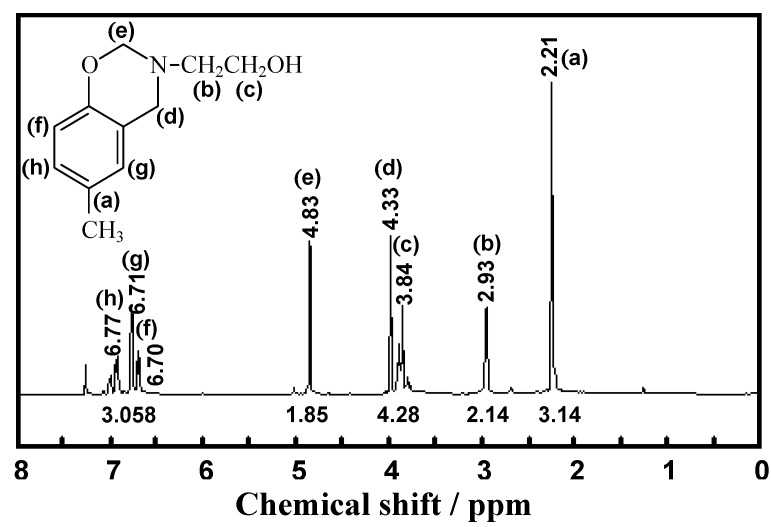
^1^H-NMR spectrum of **BM1**.

The^ 1^H-NMR spectrum of **BM1** (Figure 2) exhibits two characteristic peaks corresponding to the two methylene groups in the oxazine ring, specifically at δ_H_ = 4.33 ppm for the one between benzene and nitrogen (N-**CH_2_**-Ar) and at δ_H_ = 4.83 ppm for the other between nitrogen and oxygen (O-**CH_2_**-N). 

In addition, the chemical shifts of the methylene protons of the substituted group at nitrogen were found at 2.93 ppm (adjacent to nitrogen, N-**CH_2_**-CH_2_-OH) and at 3.84 ppm (adjacent to the hydroxyl group, N-CH_2_-**CH_2_**-OH). 

**Figure 3:**
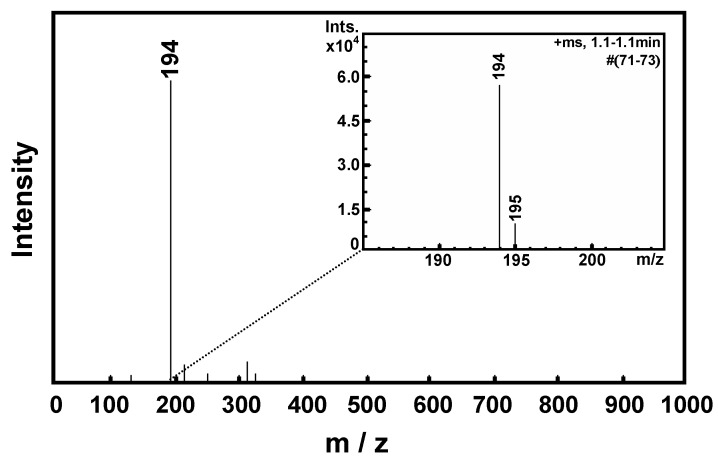
Mass spectrum of **BM1**.

A soft ionization mass spectrometer was used to determine the molecular weight of **BM1 **from the molecular ion peak. The calculated molecular weight of **BM1** was found to be 193. Figure 3 shows only one peak occurring at m/z = 194, which was assigned to the protonated peak (M+1) of **BM1**. By expanding the chemical shift scale, a small satellite peak due to ^13^C atoms in the **BM1**molecule was found at m/z = 195 (Figure 3) since the percent abundance of ^13^C atoms is found to be 1.1% of all carbon atoms. From all the characterization results, it can be concluded that the product of *p*-cresol, paraformaldehyde, and ethanolamine from the Mannich reaction is 3,4-dihydro-3-(2’-hydroxy ethylene)-6-methyl-2H-benzoxazine (**BM1**).

By similar characterizations, the products, **BM2** and **BM3**, were identified as 3,4-dihydro-3-(2’-hydroxyethylene)-6-ethyl-2H-benzoxazine and 3,4-dihydro-3-(2’-hydroxy ethylene)-6-methoxy-2H-benzoxazine, respectively. It should be noted that the Mannich reaction gives the benzoxazine monomer as a single product, indicating that the reaction is a simple and effective reaction for monomer preparation. The characterization of all products is summarized in the Experimental section and the molecular structures are shown in scheme 1. 

### 2.2. Liquid Extraction of Alkali Metal Ions

**Figure 4:**
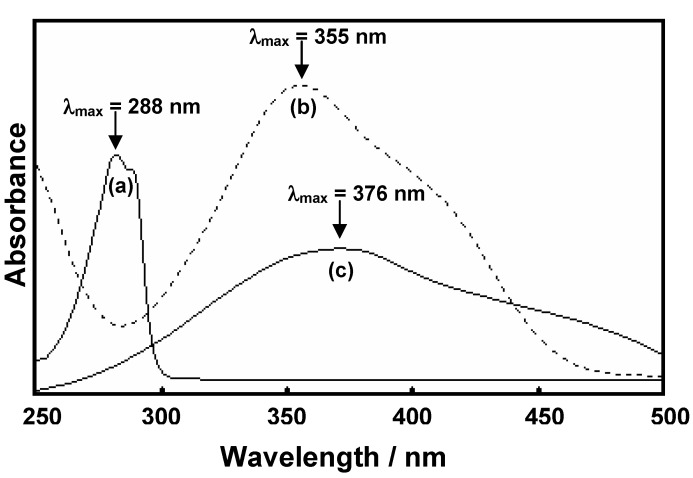
UV-Vis absorption spectra of (a) **BM1**, (b) Na^+^-picrate, and (c) complex of **BM1** and Na^+^ ions.

To clarify the relationship between the structure of products (**BM1**, **BM2**, and **BM3**) and alkali metal ion extraction ability, the qualitative and quantitative analyses were performed using Pedersen’s technique [16,17]. Figure 4 compares the absorption spectra of **BM1**, Na^+^-picrate and the extracted Na^+^-**BM1** complex in organic phase. On complexation, the aromatic π→π* transition of **BM1** [**λ**_max_ = 288 nm, Figure 4(a)] disappeared and the new absorption band [**λ**_max_ = 376 nm, Figure 4(c)] which was slightly shifted from that of Na^+^-picrate [**λ**_max_ = 355 nm, Figure 4(b)] was observed. This implied that Na^+^ ion diffused from aqueous Na^+^-picrate to form Na^+^-**BM1** complex in CH_2_Cl_2_ phase.

The maximum absorption bands of all benzoxazine derivatives (**BM1**, **BM2** and **BM3**) and their alkali metal ion picrate as well as the corresponding metal benzoxazine complexes are similar and summarized in Table 1.

**Table 1:** Maximum absorption peaks of benzoxazine derivatives, alkali – picrates, and alkali metal ion - benzoxazine complexes.

Benzoxazine derivatives	λ_max _(nm)	M^+^-picrates	λ_max _(nm)	M^+^-benzoxazine derivative complexes, λ_max_ (nm)		
				Na^+^	K^+^	Cs^+^
**BM1**	288	**Na^+^-picrate**	355	376	432	422
**BM2**	288	**K^+^-picrate**	355	378	435	430
**BM3**	298	**Cs^+^-picrate**	355	396	450	445

**Figure 5:**
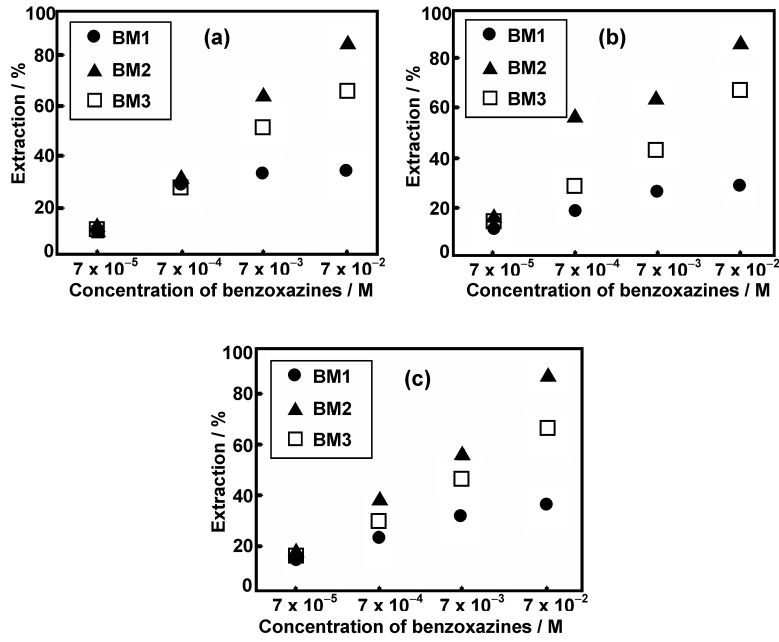
Extraction percentages of 7.0 × 10^−5^ M alkali-picrate (**a**) Na^+^- picrate, (**b**) K^+^-picrate, and (**c**) Cs^+^-picrate by various concentrations of benzoxazine derivatives in CH_2_Cl_2_ at 25°C.

With increasing concentrations of **BM1**, **BM2**, and **BM3**, the extraction percentage of all alkali metal ions gradually increases, as presented in Figure 5. The dependence of the extraction ability on benzoxazine monomer concentrations exhibits supramolecular properties like the benzoxazine dimers reported elsewhere [10]. For all concentrations, the extraction ability is **BM2** >** BM3** > **BM1**. This implies that substituent at the phenyl ring of benzoxazine monomer is responsible for the formation of the supramolecular assembly to form the space available to entrap the alkali metal ions for complexation.

**Figure 6:**
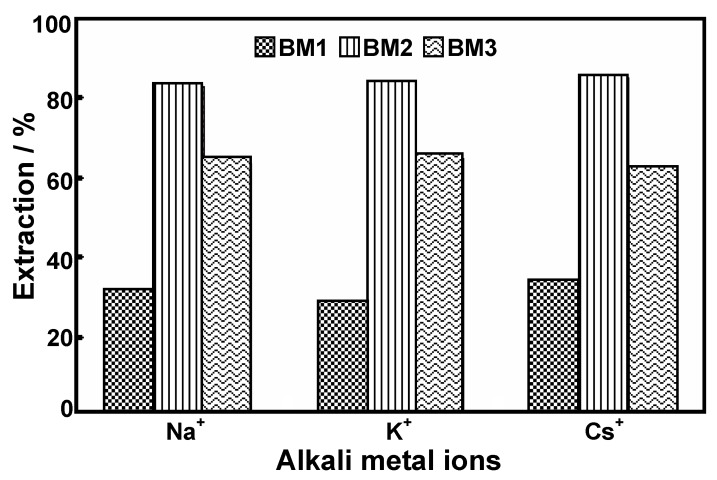
Extraction percentages of 7.0 × 10^−5^ M alkali metal ions by 7.0 × 10^−2^ M benzoxazine derivatives in CH_2_Cl_2_ at 25°C.

At the highest concentration of benzoxazine (7.0 × 10^−2^ M), the ion extraction % of the individual benzoxazines for each alkali ion is not significantly different; approximately 30%, 80%, 60% of Na^+^, K^+^, Cs^+^ were extracted by **BM1**, **BM2** and **BM3**, respectively, as shown in Figure 6. 

The highest efficiency of **BM2** for different sizes of alkali metal ions demonstrated that the generated molecular assembly provides a space for entrapping alkali metal ions. In addition, the electron donating group, –C_2_H_5_ substituted at the *para*-position also enhanced the electrostatic interaction of guest (Na^+^, K^+^, Cs^+^), consequently, alkali metal ions-**BM2** host-guest compounds with high yield were formed. Observation of lower extraction via molecular assembly might be due to the electron withdrawal from the –OCH_3 _group of **BM3**. Additionally, the lowest extraction ability found for **BM1** suggested a reduction of available space in the molecular assembly because of the least sterichindrance of substituent group –CH_3 _in **BM1** compared to –C_2_H_5_ and –OCH_3_ in **BM2** and **BM3**, respectively.

### 2.3. Complexation of Benzoxazine Monomers and Ce(III) Ion

**Figure 7:**
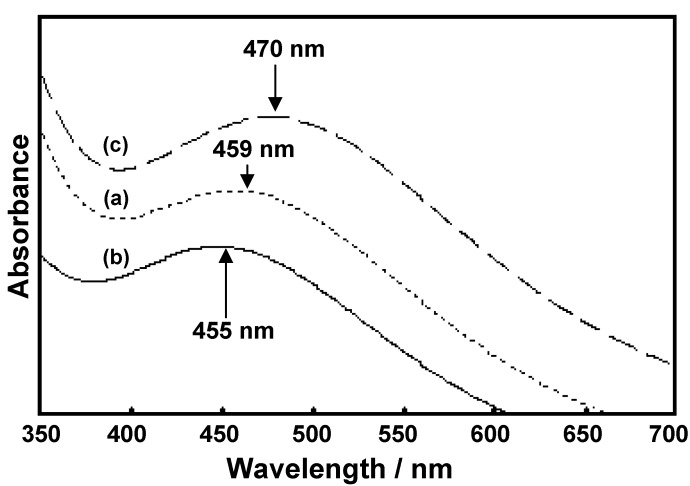
UV-Vis absorption spectra of complexes in ethanolic solution:Ce(III)-**BM1**, (b)Ce(III)-**BM2**, and (c) Ce(III)-**BM3**.

The complex formation between Ce(III) ion and benzoxazine monomers showed λ_max_ at 459, 455, and 470 nm for Ce(III)-**BM1**, Ce(III)-**BM2**, and Ce(III)-**BM3**, respectively, and their absorption spectra are illustrated in Figure 7.

**Figure 8:**
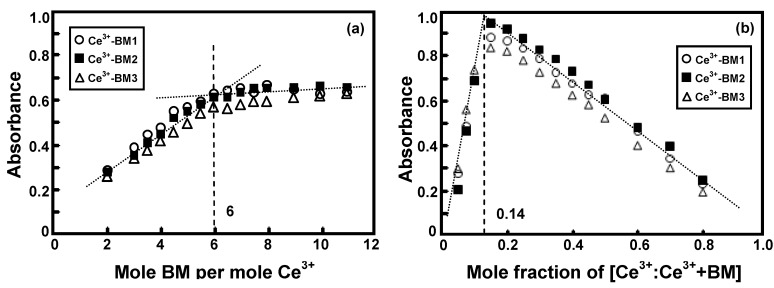
(**a**) Molar ratio plot and (**b**) Job’s plot of Ce(III)-benzoxazine monomer complexesin ethanolic solution.

Figure 8 shows the molar ratios and Job’s plots of the complexes in ethanol. 

The results obtained from the molar ratio and the Job’s methods are clearly in good agreement with Ce(III) : benzoxazine monomers (**BM1-BM3**) ratios of 1:6. This shows that the *para*-substituted groups on the molecular structure of benzoxazine monomers did not effect the Ce(III)-ligand ratio.

**Figure 9:**
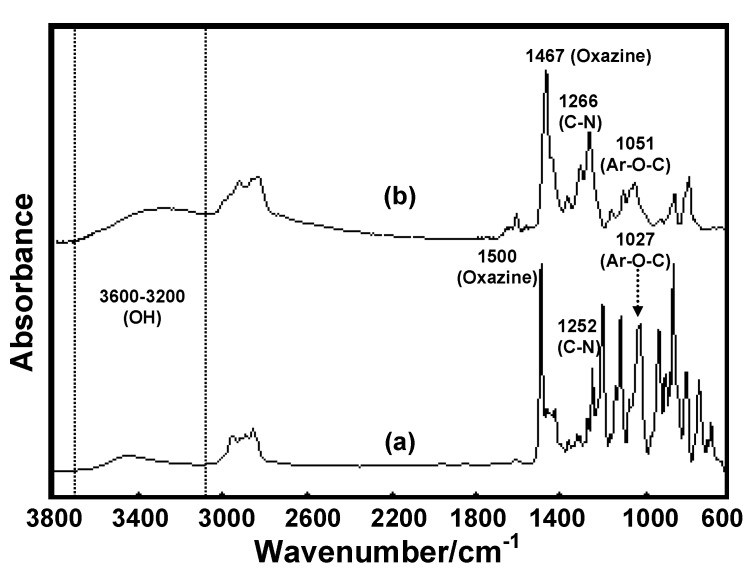
FTIR spectra of (a) **BM1** and (b) Ce(III)-**BM1** complexes.

To clarify the complex formation evidence, the FTIR spectrum of **BM1** [Figure 9(a)] and that of the complex between **BM1** and Ce(III) ion [Figure 9(b)] were compared. It was found that the characteristic peaks of **BM1** [oxazine ring, C-N stretching and aromatic ether (Ar-O-C)] were shifted from 1500, 1252 and 1027 cm^−1^ to 1467, 1266 and 1052 cm^−1^, respectively. This implied that the benzoxazine monomer might form a complex with Ce(III) ion by using an oxygen or nitrogen atom in the oxazine ring as coordination atoms. However, the hydroxyl peak in the range of 3600–3200 cm^−1^ was unchanged. Since the complex is (unhygroscopic) dried by anhydrous Na_2_SO_4_ and vacuum evaporated, this might be due to the fact that the hydroxyl group of **BM1** was not involved in the complex formation. This evidence was also confirmed by the other related works, since benzoxazine monomers without a hydroxyl group can form complexes with Ce(III) ion.

### 2.4. Computational Simulation

**Figure 10:**
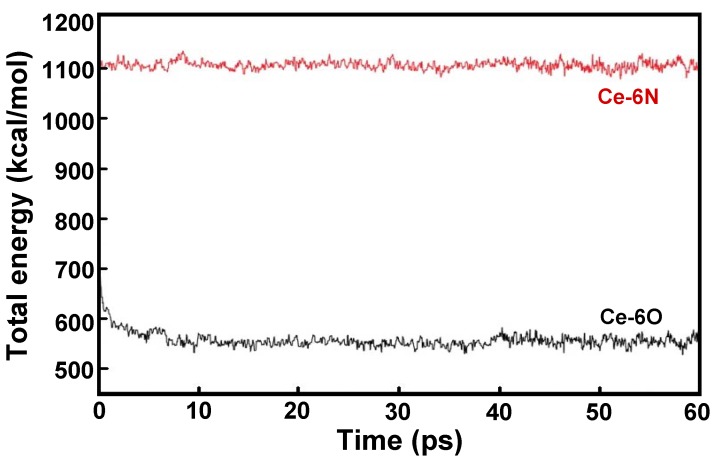
Graph of potential energy *vs.* time during geometry optimization.

To propose the possible structure of Ce(III)-**BM1** complex, a computational simulation was studied. By using electrostatic interaction of Ce(III) with the electron donor atom, O or N of **BM1** ligand, the lowest energy for equilibration was found with in 60 ps simulation time, as shown in Figure 10. The total energy profile indicated equilibration of only 10 ps.

**Figure 11:**
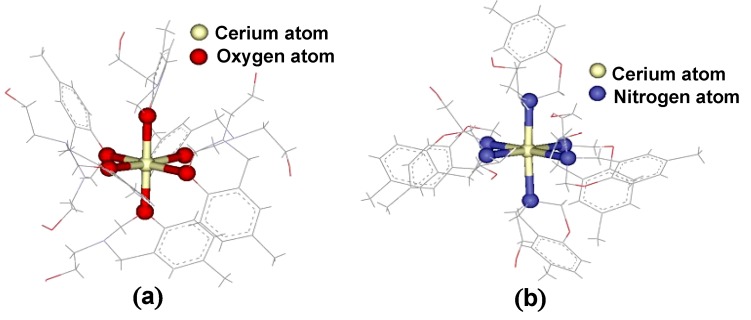
Orientation of (**a**) Ce-O and (**b**) Ce-N complexes.

The structure of the cerium complex binding with the O position was of lower energy than binding to the N-atom (Figure 11). It is indicated that the Ce(III) ion showed specific binding to the O-atom of the **BM1 **ligand along the octahedral structure during the optimization using MD simulations, Figure 11(a). As expected, the Ce(III) ion was attracted to the O-atom of the octahedral with a stable trajectory. This geometry proved that at room temperature Ce(III) ion specifically binds to the O-atom of **BM1** molecule in octahedral complex.

**Figure 12:**
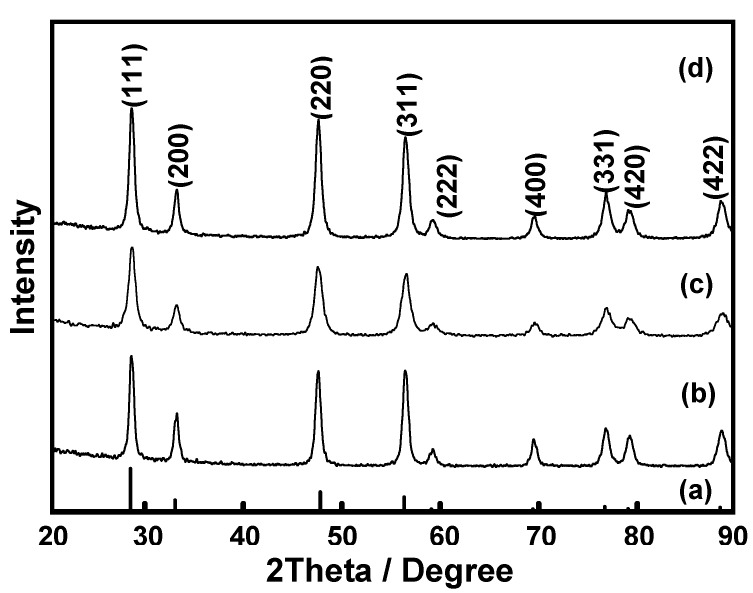
XRD patterns of (a) JCPDS No. 34-0394 and ceria powder derived from Ce(III)-benzoxazine monomer complexes: (b) Ce(III)-**BM1**, (c) Ce(III)-**BM2**, and (d) Ce(III)-**BM3**.

To recover the expensive Ce(III) ions after the complex formation study, the obtained Ce(III)-benzoxazine monomer complexes were collected, the solvent removed and then calcined at 600 °C for 2 h [19]. The XRD diffraction patterns of the calcined powders derived from all of theCe(III)-benzoxazine monomer complexes show the reflection peaks which are in agreement with the fluorite structure, CeO_2 _(JCPDS No. 34-0394), as shown in Figure 12. The crystallite size of the CeO_2_ produced showed no significant difference (Table 2).

**Table 2:** The crystallite sizes of CeO_2_ determined by the application of the Scherrer equation.

Ce(III)-benzoxazine monomer complexes	Ce(III)-BM1	Ce(III)-BM2	Ce(III)-BM3
**Crystallite size (nm)**	16	11	14

**Figure 13:**
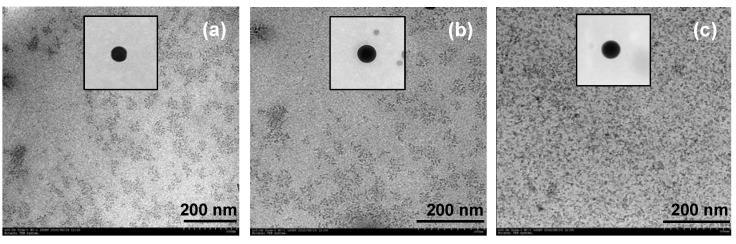
TEM micrographs at 100 kv of ceria nanoparticles derived from Ce(III)-benzoxazine complexes (**a**) Ce(III)-**BM1** (×20.0 k), (**b**) Ce(III)-**BM2 **(×24.0 k) and (**c**) Ce(III)-**BM3** (×25.0 k).

By measuring the particle size from TEM micrographs, most particles obtained from all complexes were found to be spherical, with an average diameter of 10–20 nm (Figure 13). In addition, the results also indicate that the type of benzoxazine monomer used does not affect the morphology and the particle size of the ceria.

## 3. Experimental

### 3.1. Chemicals

Paraformaldehyde was obtained from Sigma (USA). 4-Methoxyphenol, 4-ethylphenol, *p*-cresol, ethanolamine, potassium hydroxide, cesium carbonate and sodium sulfate anhydrous were ordered from Fluka Chemicals (Buchs, Switzerland). Cerium(III) nitrate hexahydrate [Ce(NO_3_)_3_ 6H_2_O, 99.50% purity] was bought from Acros Organics. Ethanol, iso-propanol, acetonitrile, sodium hydroxide, picric acid, and methylene chloride, diethylether were the products of Ajax Chemicals (Australia). All chemicals were analytical grade and used as received.

### 3.2. Instruments

The obtained products were characterized with a Fourier transform ^1^H-NMR spectrometer (Varian Mercury-400 spectrometer) with CDCl_3_ as a solvent. The infrared spectra were obtained from a Fourier transform infrared spectrophotometer (Bruker ALPHA FT-IR spectrometers) with 32 scans at a 4 cm^−1^ resolution. The mass spectra of products dissolved in methylene chloride were obtained on an ESI-MS instrument (Bruker Esquire mass spectrometer). The UV-Visible spectra and the absorption of all mixed solutions were measured with a Shimadzu UV-1700 spectrophotometer over a wavelength of 200 to 700 nm. 

The calcined powders were investigated by X-ray diffraction (XRD: a Bruker D8-Advance X-ray diffractrometer with CuK_α_ radiation). Diffraction patterns were recorded over a range of 2θ angles from 20 to 90 degrees in a step-scanning mode (0.02° steps with a step-counting time of 2 sec). The crystalline phase was identified from the Joint Committee on Powder Diffraction Standard (JCPDS) file No. 34-0394.

The crystallite size (D_XRD_) of the calcined powders was determined using the Scherrer equation: D = 0.9λ/βcosθ, where λ is the wavelength of the X-rays (1.5406 Å), θ is the scattering angle of the main reflection (111), and β is the corrected peak at full width at half-maximum (FWHM) intensity.

The morphology of the obtained ceria powders observed with a transmission electron microscope (TEM) was taken at an accelerating voltage current of 100.0 kV by Hitachi H-7650 (Hitachi High-Technology Corporation, Japan).

### 3.3. Preparation of Monomers by the Mannich Reaction

3,4-Dihydro-3-(2’-hydroxyethylene)-6-methyl-2H-benzoxazine (**BM1**) was prepared as shown in Scheme 1. Paraformaldehyde (6.31 g, 210.0 mmol) was dissolved in dioxane (50.0 mL), followed by the addition of ethanolamine (6.0 mL, 100.0 mmol) in dioxane (10.0 mL) and *p*-cresol (10.8 g, 100.0 mmol) in dioxane (50.0 mL). The mixture was stirred and allowed to reflux for 3 h. The solution was then collected to remove dioxane by vacuum distillation. The yellow and sticky liquid phase obtained was dissolved in diethyl ether. The resulting solution was then extracted with 3 N NaOH (20 mL) three times, followed by several water washes. The product was dried over anhydrous sodium sulfate and the solvent was removed to obtain a clear and yellow liquid product. Yield 75%; clear and yellow liquid; R_f _= 0.82 (10% MeOH in CH_2_Cl_2_); FTIR (ATR, ZnSe, cm^−1^): 3600–2900 (br, OH), 2877 (s, CH), 1500 (oxazine ring), 1226 (s, CN), 1126 (s, CO), 1027 (s, Ar-O-C), 817 (b, CH); ^1^H NMR (400 MHz, CDCl_3_, ppm): δ_H_ 2.21 (3H, s, Ar-**CH_3_**), 2.93 (2H, t, **CH_2_**CH_2_OH *J_2 _*= 3.11 Hz), 3.84 (2H, t, CH_2_**CH_2_**OH* J_2 _*= 3.11 Hz), 4.33 (2H, s, Ar-**CH_2_**-N), 4.83 (2H, s, O-**CH_2_**-N), 6.70 (H, d, **Ar-H**, *J_3_* = 2.34 Hz), 6.71. (H, s, **Ar-H**), 6.77 (H, d, **Ar-H**, *J_3 _*= 2.34 Hz). ESI-MS (m/z): 194 (M+1).

Similarly, 3,4-dihydro-3-(2’-hydroxyethylene)-6-ethyl-2H-benzoxazine (**BM2**) and 3,4-dihydro-3-(2’-hydroxyethylene)-6-methoxy-2H-benzoxazine (**BM3**) were prepared as described for **BM1**. The starting materials for **BM2** and **BM3 **were 4-ethylphenol (12.22 g, 100.0 mmol) and 4-methoxyphenol(12.41 g, 100.0 mmol), respectively (Scheme 1). 

**BM2**: yield 72%; clear and yellow liquid; R_f_ = 0.80 (10% MeOH in CH_2_Cl_2_); FTIR (ATR, ZnSe, cm^−1^): 3600–2900 (br, OH), 2954 (s, CH), 1496 (oxazine ring), 1226 (s, CN), 1126 (s, CO), 1025 (s, Ar-O-C), 825 (b, CH);^1^H NMR (400 MHz, CDCl_3_, ppm): δ_H_ 1.20 (3H, t, Ar-CH_2_**CH_3_** *J_1 _*= 7.55 Hz), 2.55 (2H, q, **CH_2_**-CH_3_* J_1 _*= 7.55 Hz), 2.96 (2H, t, **CH_2_**CH_2_OH* J_2 _*= 5.18 Hz), 3.86 (2H, t, CH_2_**CH_2_**OH* J_2 _*= 5.18 Hz), 4.34 (2H, s, Ar-**CH_2_**-N), 4.84 (2H, s, O-**CH_2_**-N), 6.70 (H, d, **Ar-H**, *J_3_* = 2.34 Hz), 6.72. (H, s, **Ar-H**), 6.77 (H, d, **Ar-H**, *J_3 _*= 2.34 Hz). ESI-MS (m/z): 208 (M+1).

**BM3**: yield 75%; clear and yellow liquid; R_f_ = 0.78 (10% MeOH in CH_2_Cl_2_); FTIR (ATR, ZnSe, cm^−1^): 3600–2900 (br, OH), 2947 (s, CH), 1496 (oxazine ring), 1211 (s, CN), 1141 (s, CO), 1027 (s, Ar-O-C), 810 (b, CH); ^1^H NMR (400 MHz, CDCl_3_, ppm): δ_H_ 2.94 (2H, t, **CH_2_**CH_2_OH * J_2_* = 4.00 Hz), 3.73 (3H, s, **CH_3_**-O), 3.77 (2H, t, CH_2_**CH_2_**OH* J_2 _*= 4.00 Hz), 4.33 (2H, s, Ar-**CH_2_**-N), 4.81 (2H, s, O-**CH_2_**-N), 6.71 (H, d, **Ar-H**, *J_3_* = 3.88 Hz), 6.49. (H, s, **Ar-H**), 6.76 (H, d, **Ar-H**, *J_3 _*= 3.88 Hz). ESI-MS (m/z): 210 (M+1).

### 3.4. Liquid Extraction of Alkali Metal Ions

Alkali metal ion extractions were qualitatively and quantitatively studied with Pedersen’s technique [16,17]. The benzoxazine monomers (**BM1**, **BM2**, and **BM3**) in methylene chloride were prepared at concentrations of 7.0 × 10^−2^, 7.0 × 10^−3^, 7.0 × 10^−4^, and 7.0 × 10^−5^ M, while the alkali metal picrates aqueous solutions (Na^+^, K^+^, Cs^+^-picrates) were prepared at a concentration of 7.0 × 10^−5^ M. Five milliliters of each solution were vigorously mixed and left at room temperature until each phase was completely separated. The absorbance of metal picrate aqueous solutions was measured by UV-Vis at λ_max_ 355.0 nm. The percentage extraction of metal concentration was calculated by the equation [(A_o_ − A) / A_o_] × 100 where A_o_ is the initial absorbance of the picrate solution, and A is the absorbance of the picrate solution after extraction with the monomer. The complexes in the organic phase were collected to clarify the ion-monomer interaction by UV-Vis. 

### 3.5. Complexation of Benzoxazine Monomers and Ce(III) Ion

The ethanolic solutions of Ce(NO_3_)_3_.6H_2_O and benzoxazine monomer derivatives (**BM1**, **BM2**, and **BM3**) were individually prepared with an equimolar concentration of 7.0 × 10^−3^ M. To investigate the complex formation, the molar ratio and the Job’s methods were applied.

For the molar ratio method, a series of solutions containing 0.50 mL of Ce(III) ion and each benzoxazine monomer (**BM1**, **BM2**, or **BM3**) of different volumes (2.00, 3.00, 3.50, 4.00, 4.50, 5.00, 5.50, 6.00, 6.50, 7.00, 7.50, 8.00, 9.00, 10.00 and 11.00 mL) was mixed and subsequently adjusted with ethanol to attain a total volume of 25.00 mL.

For the Job’s method, a series of mixture solutions of Ce(III) ion and each benzoxazine monomer (**BM1**, **BM2**, or **BM3**) with various Ce(III) ion mole fractions, X (X = 0.05, 0.08, 0.10, 0.15, 0.20, 0.25, 0.30, 0.35, 0.40, 0.45, 0.50, 0.60, 0.70 and 0.80) was prepared. 

The absorbance of each mixture solution was measured at λ_max_ 459.5, 455.5 and 470.0 nm for Ce(III)-**BM1**, Ce(III)-**BM2**, and Ce(III)-**BM3**, respectively.

All solutions of Ce(III)-benzoxazine monomer complexes were collected and dried over anhydrous sodium sulfate to eliminate water and any moisture in the solvent. The solvent was then removed by vacuum distillation to obtain the brown solid products which were collected to analyses the metal ion-monomer interaction with FTIR. 

### 3.6. Computational Simulation

Molecular dynamics (MD) simulations were conducted to investigate complementary insights into Ce(III)-**BM1** complex associations that abet the interpretation of experimental spectroscopic data. Molecular dynamics simulations were performed with the Forcite module in the Accelrys Inc Materials Studio (version 5.5) [21]. The all-atom, force-field, Universal force field was used to perform simulations of the cerium interactions of metal cations with O and N positions of **BM1** ligand, whereby the force field was used to describe all atoms in the simulated systems, including those of cerium metal cations [22,23,24,25]. Universal is moderately accurate for predicting geometries and conformational energy differences of organic molecules, main-group inorganics, and metal complexes. It is recommended for organometallic systems and other systems for which other force fields do not have parameters. The energy minimization and annealing MD simulations were performed to push the system into a global energy minimum, followed by a final MD step from which energy and structural data were retrieved after examination of the total potential energy as a function of time indicated equilibration. This final MD step was carried out in the canonical ensemble (constant number of atoms (N), simulation cell volume (V), and temperature (T)) for up to 60 picoseconds (ps) (time step 1.0 femtoseconds [fs]) using a canonical thermostat algorithm to control temperature at 298 K. 

### 3.7. Preparation of Ceria (CeO_2_) from Ce(III)-Benzoxazine Monomer Complexes

To obtain CeO_2_ powders, all complex products were calcined in alumina crucibles at 600 °C for 2 h [19]. The powders obtained were studied by XRD for phase identification and TEM for morphology observation.

## 4. Conclusions

The present work shows that benzoxazine monomers provide ion extraction properties for various ions via their molecular assembly. None of the benzoxazine monomers with different substituted groups on the phenolic moiety showed significant differences in ion extraction ability and no ion selectivity was observed. This might be due to a loose and flexible structure formation of the molecular assembly, which leads to high capability and selectivity with ion interaction. 

This study also revealed that all of the proposed benzoxazine monomers show properties as novel ligands for the Ce(III) ion. With the molar ratio and Job’s methods, the metal-ligand ratios between the Ce(III) ion and the benzoxazine monomers (**BM1**, **BM2**, and **BM3**) in ethanolic solution were found to be 1 to 6. A possible structure of Ce(III)-**BM1** complex based on molecular dynamics simulations was proposed and found to be in agreement with the FTIR results. 

Pure ceria nanoparticles were successfully prepared from the complexes of Ce(III)-benzoxazine monomers by calcinating at 600 °C for 2 h. The obtained particles were spherical with an approximate size of 10–20 nm. By the application of the aforementioned method, diverse advantages are evident, such as a simple reaction which occurs in the absence of specific solvents at room temperature. Additionally, high surface area ceria are obtained as compared with previous ligands like triethanolamine and benzoxazine dimer [18,19,20]. We plan to use the so obtained ceria nanoparticles as the solid support for metal catalysts in further studies by our research group.
